# The Antiproliferative and Apoptotic Effects of Sirtinol, a Sirtuin Inhibitor on Human Lung Cancer Cells by Modulating Akt/*β*-Catenin-Foxo3A Axis

**DOI:** 10.1155/2014/937051

**Published:** 2014-08-12

**Authors:** Yao Fong, Yin-Chieh Lin, Chang-Yi Wu, Hui-Min David Wang, Li-Li Lin, Han Lin Chou, Yen-Ni Teng, Shyng-Shiou Yuan, Chien-Chih Chiu

**Affiliations:** ^1^Department of Thoracic Surgery, Chi-Mei Medical Center, Tainan 710, Taiwan; ^2^Department of Biotechnology, Kaohsiung Medical University, Kaohsiung 807, Taiwan; ^3^Department of Biological Sciences, National Sun Yat-sen University, 70 Lien Hai Road, Kaohsiung 804, Taiwan; ^4^Department of Fragrance and Cosmetics Science, Kaohsiung Medical University, Kaohsiung 807, Taiwan; ^5^Department of Medical Research, Kaohsiung Medical University Hospital, Kaohsiung Medical University, Kaohsiung 807, Taiwan; ^6^Department of Biological Sciences and Technology, National University of Tainan, Tainan 700, Taiwan; ^7^Translational Research Center, Cancer Center, Department of Medical Research, and Department of Obstetrics and Gynecology, Kaohsiung Medical University Hospital, Kaohsiung Medical University, Kaohsiung 807, Taiwan

## Abstract

Sirtuins, NAD^+^-dependent deacetylases, could target both histones and nonhistone proteins in mammalian cells. Sirt1 is the major sirtuin and has been shown to involve various cellular processes, including antiapoptosis, cellular senescence. Sirt1 was reported to be overexpressed in many cancers, including lung cancer. Sirtinol, a specific inhibitor of Sirt1, has been shown to induce apoptosis of cancer cells by elevating endogenous level of reactive oxygen species. In the study, we investigated the effect of sirtinol on the proliferation and apoptosis of nonsmall cell lung cancer (NSCLC) H1299 cells. The results of proliferation assay and colony formation assay showed the antigrowth effect of sirtinol. The annexin-V staining further confirmed the apoptosis induction by sirtinol treatment. Interestingly, the levels of phosphorylated Akt and *β*-catenin were significantly downregulated with treating the apoptotic inducing doses. On the contrary, sirtinol treatment causes the significantly increased level of FoxO3a, a proapoptotic transcription factor targeted by Sirt1. These above results suggested that sirtinol may inhibit cell proliferation of H1299 cells by regulating the axis of Akt-*β*-catenin-FoxO3a. Overall, this study demonstrates that sirtinol attenuates the proliferation and induces apoptosis of NSCLC cells, indicating the potential treatment against NSCLC cells by inhibiting Sirt1 in future applications.

## 1. Introduction

Cancer is one of the leading causes of death worldwide. Lung cancer has higher mortality than other cancers in both men and women [1]. More than 80% of lung cancer patients are nonsmall cell lung cancer (NSCLC). NSCLC can be classified by the location, including adenocarcinoma, squamous cell carcinoma, and large cell lung carcinoma [2, 3]. The treatment options for patients with NSCLC include surgery, radiation therapy, chemotherapy, and targeted therapy. Chemotherapy is given as main treatment for more advanced cancers or for patients who are not healthy enough for surgery [4]. The chemotherapy for NSCLC treatment is developed and improved recently. However, the poor prognosis and drug resistance contribute to the low survival rate of NSCLC patients [5, 6].

Sirtuin (Sirt) family is a class III HDAC. In yeast, sirtuin protein plays an important role for lifespan extension in response to metabolic and other environmental stresses [7–10]. SIRT family proteins also target various nonhistone proteins including structural protein, signal intermediates, and transcription factors [11]. At least seven homologues (Sirt1–7) have been identified in mammalian cells [12–14]. Among Sirt proteins, the physiological role of Sirt1 in mammalian cells has been reported to prevent tumorigenesis and ensure cellular longevity, and the continuous prevention of apoptosis may induce the tumorigenesis [15]. Previous studies showed that Sirt1 overexpression in cancer correlates with silencing of tumor suppressor genes [16].

Akt is a serine/threonine kinase which has been shown to be associated with proliferation and survival [17]. The overexpression and constitutively activation of Akt was observed in many cancers, including lung cancer [6] and breast cancer [5] cells, which are highly correlated with the chemoresistance of cancer cells. On the contrary, Forkhead box O3a (FoxO3a), a transcription factor, is the downstream target of Akt [17]. FoxO3a can promote anticell growth or apoptosis signaling through either inducing expression of the proapoptotic Bcl-2 such as Bim [18], stimulating the expression of death receptor ligands, including Fas ligand and tumor necrosis factor-related apoptosis-inducing ligand (TRAIL), or increasing the protein levels of cyclin-dependent kinase inhibitors [19]. Aberrant Akt-mediated phosphorylation of FoxO3a causes the survival and proliferation of cancer cells. Therefore, the signaling axis of Akt and FoxO3a regulates cell growth and survival may shed the light on developing a promising strategy for lung cancer treatment [17, 18, 20, 21].

The tumor suppressor forkhead family of transcription factors (FOXO) is one of SIRT1 substrate. The acetylation of FOXO3 increases in response to oxidative stress [22]. Therefore, FOXO3 could control the balance between stress resistance and apoptosis through its downstream targets such as GADD45 and Bim [23]. For example, Frazzi's work reported that resveratrol-induced apoptosis of Hodgkin lymphoma cells is involved in the inhibition of SIRT1 and the hyperacetylation of FoxO3 [24].

In this study, we demonstrated antigrowth and apoptosis-inducing effect of sirtinol on lung cancer cells. The sirtinol-induced antiproliferation and apoptosis of lung cancer via FoxO3a-Akt were investigated and discussed.

## 2. Methods

### 2.1. Reagents

Sirtinol was purchased from Calbiochem (Darmstadt, Germany). Sirtinol was dissolved in 100% DMSO at concentration of 10 mM and stored at −20°C until use. The following compounds were obtained from Gibco BRL (Maryland, USA): Dulbecco's modified eagle medium (DMEM), Ham's F-12 Nutrient Mixture (F-12) fetal bovine serum (FBS), trypan blue, penicillin G, and streptomycin. Dimethyl sulfoxide (DMSO), ribonuclease A (RNase A), and propidium iodide (PI) were purchased from Sigma-Aldrich (Missouri, USA). Antibodies against FoxO3a were obtained from Epitomics (California, USA). Annexin V-FITC staining kit was purchased from Strong Biotech (Taipei, Taiwan). Antibodies against phospho-Akt, *β*-catenin, Sirt1, and *β*-actin were purchased from Santa Cruz Biotechnology (California, USA). Anti-mouse and anti-rabbit IgG peroxidase-conjugated secondary antibodies were purchased from Pierce (Illinois, USA). The anti-rabbit Rhodamine-conjugated antibody was purchased from Abcam (Cambridge, UK).

### 2.2. Cell Culture

Human nonsmall cell lung cancer (NSCLC) cell lines H1299 were obtained from American Type Culture Collection (ATCC; Virginia, USA). All tested cells were maintained in DMEM/F-12 in 1 : 1 ratios (pH 7.4) supplemented with 10% FBS and 1% penicillin-streptomycin (100 units/mL penicillin and 100 *μ*g/mL streptomycin). All cells were incubated in a humidified atmosphere incubator containing 5% CO_2_ at 37°C.

### 2.3. Proliferation Assay

The cell proliferation of H1299 cells was determined by trypan blue dye exclusion assay [25] combined with Countess Automated Cell Counter performed according to the manufacturer's instruction (Invitrogen; California, USA). 1 × 10^5^ cells were seeded in 12-well plates and treated with indicated concentrations of sirtinol (0, 10, 20, and 50 *μ*M) for 24 h and 48 h, respectively. After incubation, the cells were stained by 0.2% trypan blue and counted by Countess.

### 2.4. Apoptosis Assessment

To examine the apoptosis assessment of H1299 cells after being sirtinol treated, Annexin-V/PI double staining [26] was performed to detect the externalization of phosphatidylserine (PS). 3 × 10^5^ cells were seeded onto a 6-well plate and incubated for 24 h and treated with different concentrations of sirtinol (0, 5, 10, 20, and 50 *μ*M) for 24 h, respectively. Afterwards, cells were harvested and stained by Annexin-V staining kit (Strong Biotech, Taipei, Taiwan) according to the manufacturer's manual. The cells were washed in 1 × Annexin-V buffer (HEPES, NaCl, CaCl_2_·2H_2_O) and stained by Annexin-V for 30 min in 37°C water bath. The cells were analyzed by flow cytometry (FACS Calibur; Becton Dickinson; California, USA). The results of apoptosis assessment were analyzed by using the FlowJo software (Treestar, Inc.; California, USA).

### 2.5. Colony Formation Assay

100 cells were planted onto a 6-well plate, and, after 24 h incubation, the cells were treated with different concentrations of sirtinol (0, 10, 20, and 50 *μ*M). After 15 days incubation, the colonies of cells were glutaraldehyde-fixed and stained with crystal violet (0.01% w/v) for 1 h. The diameter of colonies was determined by Image-Pro Plus software (Media Cybernetics; Maryland, USA).

### 2.6. Cell Cycle Analysis

3 × 10^5^ cells were seeded in 6-well plates and incubated for 24 h. The cells were treated with different concentrations of sirtinol (0, 10, 20, and 50 *μ*M) for 24 h. Then, the cells were harvested and fixed with ice-cold 75% ethanol for at least 24 h at −20°C. Ethanol-fixed cells were collected by centrifugation and washed with PBS. After centrifugation, the cells were resuspended in 500 *μ*L PBS containing 2.5 *μ*g/mL RNase A and incubated for 30 min in 37°C water bath. Then, the cells were centrifuged and stained by propidium iodide (PI) [27]. The cells were analyzed by flow cytometry. The cell cycle distribution results were analyzed by using the FlowJo software [28].

### 2.7. Western Blot Analysis

6 × 10^5^ cells were harvested and lysed with 1 × RIPA lysis buffer. Lysates were centrifuged at 13000 rpm for 30 min and the protein concentration in the supernatant was determined. 40 *μ*g of protein was resolved by 10% SDS-polyacrylamide gel electrophoresis (SDS-PAGE). Afterwards, the proteins were electrotransferred to the nitrocellulose membrane (PALL; Michigan, USA) and blocked with 5% fat-free milk in PBS-T buffer (1 × PBS containing 0.1% Tween 20) for 1 h. Then the membranes were incubated with primary antibodies against specific proteins overnight at 4°C. The blot was washed by PBS-T buffer and incubated with corresponding secondary antibodies for 90 min. The signals were detected by an enhanced chemiluminescence (ECL) detection kit (Amersham Piscataway; New Jersey, USA) [29].

### 2.8. Statistical Analysis

Differences between DMSO- and sirtinol-treated cells were analyzed in at least triplicate experiments. The significance of the differences was analyzed by Student's *t*-test, with *P* < 0.05 considered significantly.

## 3. Results

### 3.1. Sirtinol Exerts Antiproliferative Effect towards NSCLC Cells

**Figure 1:**
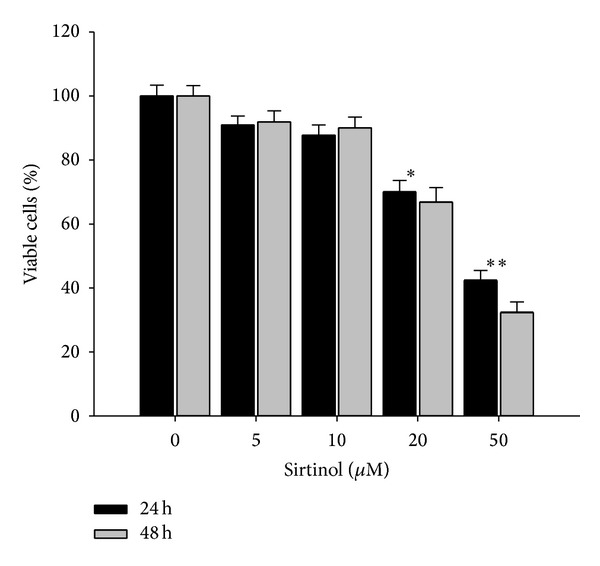
Effect of sirtinol on cellular proliferation of H1299 cells. H1299 cells treated with different concentrations (5, 10, 20 and 50 *μ*M) of sirtinol for 24 h and 48 h, respectively. The cell survival was determined by the trypan blue staining assay combined with the Countess Automated Cell Counter. **P < 0.001 against vehicle control.

**Figure 2:**
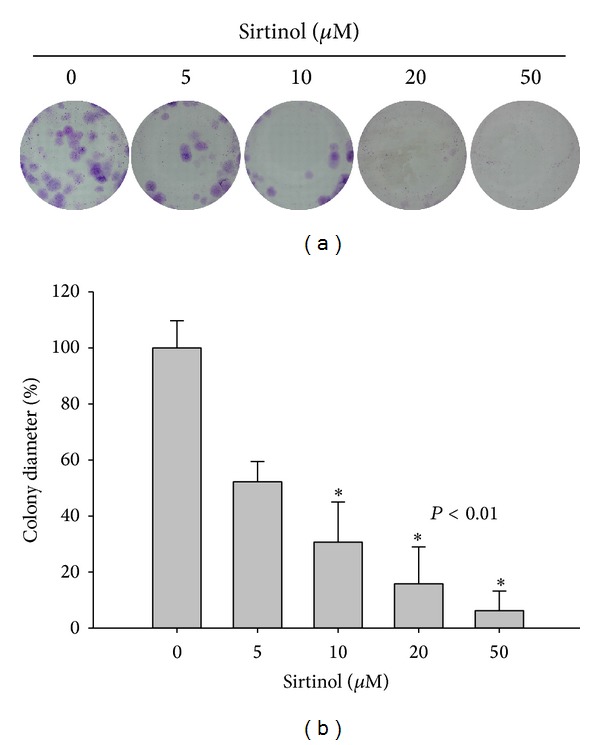
Sirtinol inhibits the colony formation of lung cancer cells. H1299 cells were treated with different concentrations (5, 10, 20 and 50 *μ*M) of sirtinol for 15 days, respectively. Afterwards, the cells were glutaraldehyde-fixed and stained with Giemsa stain for 1 h. (a) The colony formation analysis of H1299 cells. (b) The quantification analysis of the colony diameter. Data are represented as mean ± SD (*n* = 3). *P < 0.01 compared with the vehicle control.

We used sirtinol, a specific and direct inhibitor of the sirtuin class of deacetylase activity, to inhibit Sirt1 in H1299 cells [30]. To investigate the effect of sirtinol on cell proliferation, the NSCLC cell line H1299 was treated with different concentrations of sirtinol for 24 and 48 h, respectively. The cell viable cells were measured by trypan blue staining assay combined with automatic cell counter. The results of both cell proliferation assay and colony formation assay showed the antigrowth effect of sirtinol on NSCLC H1299 cells, especially at the dose of 20 and 50 *μ*M sirtinol treatment (Figures 1 and 2). We also examined whether sirtinol induced NSCLC H1299 cells apoptosis. We treated the cells with different concentrations of sirtinol (0, 10, 20, and 50 *μ*M) and then conducted the flow cytometry-based Annexin V and PI double staining assay. The cellular apoptosis was detected at high concentration of sirtinol treatment (Figure 4).

### 3.2. The Effect of Sirtinol on Regulating Cell Cycle Distribution of H1299 Cells

**Figure 3:**
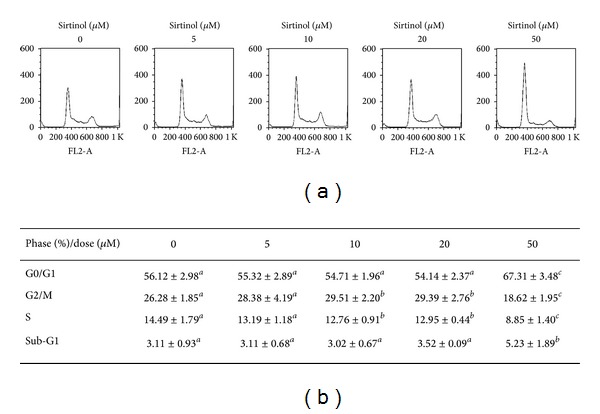
The effect of sirtinol on cell cycle distribution of lung cancer cells. H1299 cells treated with indicated concentrations (from 5 to 50 *μ*M) of sirtinol for 24 h, respectively. Cells were stained with PI and detected the cell cycle distribution by flow cytometry. (a) Flow cytometry profile represents PI staining in *x*-axis and cell number in *y*-axis. (b) The quantitative analysis of cell cycle distribution. Different letter notations indicate the statistical significance between drug treatment and vehicle (*a *versus* b* and* a *versus* c* indicate the *P* < 0.05 and 0.001, resp.).

**Figure 4:**
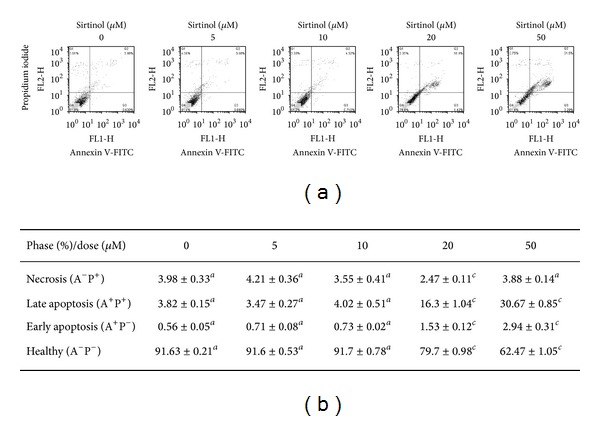
Sirtinol induces apoptosis of H1299 cells. Cells were treated with indicated concentrations of sirtinol and stained with Annexin-V and PI at 24 h, respectively. (a) Flow cytometry profiling represents the results of Annexin-V-FITC staining. (b) The quantificative analysis of cell apoptosis. Different letter notations indicate the statistical significance between sirtinol treatment and vehicle (*a *versus* b* and* a *versus* c* indicate the *P* < 0.05 and 0.001, resp.).

In previous study, Sirt1 has shown to exert the ability to induce cell cycle arrest and resistance to oxidative stress [31]. Therefore, we examined whether sirtinol induced NSCLC H1299 cell cycle disturbance. After sirtinol treatment, the cells were stained by PI, and detected the cell cycle distribution by flow cytometry (Figure 3). The result showed that the highest dose (50 *μ*M) of sirtinol treatment induces G1-phase accumulation.

### 3.3. The Effect of Sirtinol on Modulating the Expression of Prosurvival Proteins

**Figure 5:**
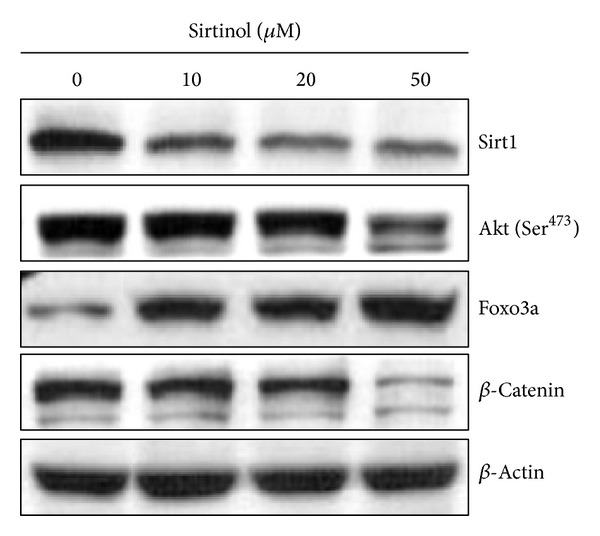
Modulation of protein levels in NSCLC H1299 cells after sirtinol treatment. H1299 cells treated with indicated concentrations (10, 20, and 50 *μ*M) of sirtinol for 24 h, respectively. The results of Western blot of Sirt1 nonhistone target protein, including FoxO3a, Akt phosphorylation, and *β*-catenin. *β*-Actin as an internal control.

Sirt1 was reported to deacetylate various nonhistone protein targets, including p53, NF-*κ*B, *β*-catenin, and FoxO3a [32–34]. Because H1299 cells do not express the tumor suppressor p53 protein, we used Western blot to analyze the protein level of *β*-catenin, NF-*κ*B p65, and FoxO3a after sirtinol treatment (Figure 5). NF-*κ*B p65 is a critical transcription factor that regulates inflammation and cell proliferation and differentiation. NF-*κ*B was reported to be aberrantly expressed and constitutively activated in lung cancer [35, 36]. However, the results of Western blot showed that no significant changes of NF-*κ*B p65 protein levels were observed (data not shown), suggesting that the antiproliferative effect of sirtinol on lung cancer H1299 cells is NF-*κ*B p65-independent. On the contrary, the previous study showed that sirt1 plays a tumor suppressive role mediated through inhibition of *β*-catenin [37]. The protein level of *β*-catenin was decreased only in cytotoxic concentrations 20 and 50 *μ*M, suggesting that *β*-catenin did not involve in Sirt1-induced cells invasiveness, and Sirt1 may play a role as a tumor promoter in H1299 cells.

The transcription factor FoxO3a has a crucial role in mediating the cytostatic and cytotoxic effects of anticancer drug [38, 39]. Recent study suggested that FoxO3a may be a major mediator for the cytotoxic effect of cisplatin in lung cancer cells [40]. More recently, Zheng's work demonstrated that ectopic expression of FoxO3a enhanced p21^CIP1/WAF1^ expression and berberine, a compound derived from traditional Chinese medicine-induced apoptosis in human lung adenocarcinoma cells [41], indicating the important role of FoxO3a in the initiation of apoptosis in cancer cells.

Furthermore, FoxO3a is a well-known nonhistone target of Sirt1. It has been reported that Sirt1 would deacetylate and repress FoxO3a activity and reduce forkhead-dependent apoptosis [23]. In the result of Western blot, FoxO3a was increased after sirtinol treatment, suggesting that Sirt1 represses the protein level of FoxO3a in NSCLC H1299 cells.

We next examined the proteins that involve in cellular proliferation and metastasis protein, including the phosphorylation of Akt [42–44] (Figure 5). The phosphorylation of levels of Akt was decreased only in cytotoxic concentrations 20 and 50 *μ*M, suggesting that Akt did not involve in sirtinol-induced inhibition of cells' invasiveness but the apoptosis pathway.

## 4. Discussion

Sirt1 has been shown to be involved in a variety of biological processes, including transcriptional silencing, stress responses, aging, apoptosis, tumorigenesis, and cellular metabolism [45, 46]. It targets diverse histone and various nonhistone proteins including structural protein, signal intermediates, and transcription factors, such as *α*-tubulin, p53 [47–49], FoxO [20, 23, 31], E2F1 [50], NF-*κ*B [51, 52], and Ku70 [53]. Additionally, Sirt1 has been shown to overexpress in several cancer cells, including breast, prostate, ovarian, and colon cancer cell lines [46, 54–58]. Recent studies showed the tumorigenicity role of Sirt1. For example, Sirt1 was reported to promote cell migration and invasion of prostate cancer [59, 60]. Consistently, our preliminary result also showed that Sirt1 would overexpress in NSCLC cell lines (data not shown).

The production of ROS has been resulted in cellular damage and genomic instability [61]. Furthermore, many anticancer drugs could induce apoptosis by increasing the level of endogenous ROS in the cancer cells [62, 63]. Recent study showed that sirtinol treatment induces the apoptosis of lung carcinoma 549 cells by increasing the level of endogenous ROS [64].

**Figure 6:**
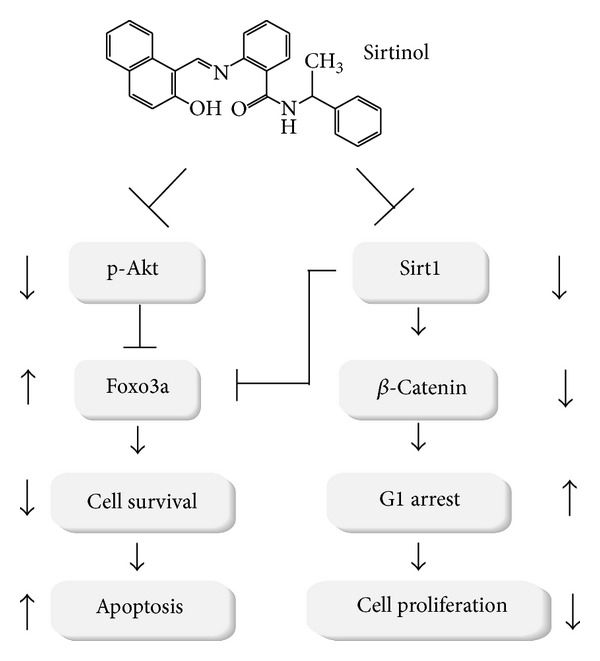
Possible model of sirtinol-induced antiproliferation and apoptosis in lung cancer cells. Sirtinol downregulates the activation of prosurvival Akt serine/threonine kinase and the protein level of *β*-catenin, a proliferation-associated transcription factor, insulting in the cell cycle G_1_-phase accumulation and the growth arrest. On the contrary, sirtinol treatment causes the upregulation of the proapoptotic transcription factor FoxO3a, a target of both Akt signaling and Sirt1. This may render H1299 cells more sensitive to apoptosis. Finally, sirtinol induces the apoptosis of lung cancer cells.

Accordingly, we proposed the positive role of Sirt1 in the progression of NSCLC cells. We used sirtinol, a specific inhibitor of sirtuin, to inhibit Sirt1. Our results showed that sirtinol exerts a significant cytotoxicity towards NSCLC H1299 cells with a dose-responsive manner (20 and 50 *μ*M). The Western results showed that sirtinol-induced inhibition of Sirt1 resulted in the increased transcription factor FoxO3a. These above results suggest a close correlation of sirtinol-induced antilung cancer and the regulation of Akt-FoxO3a signaling pathway (Figure 6).

## 5. Conclusions

In this study, we used sirtinol, a specific inhibitor of Sirt1, to investigate the role of Sirt1 in the apoptosis and proliferation of H1299 cells. Sirtinol treatment causes the cell cycle arrest and apoptosis of lung cancer cells. Furthermore, sirtinol-induced inhibition of Sirt1 activity may increase the protein levels of the transcription factor Foxoa3a whereas it downregulates the activation of Akt and the protein level of *β*-catenin after sirtinol treatment. Our recent work suggests that sirtinol-induced antiproliferation and apoptosis of lung cancer cells may be correlated with Akt-FoxO3a signaling pathway (Figure 6).
